# RNA Sequencing and Cell Models of Virus-Associated Cancer (Review)

**DOI:** 10.17691/stm2022.14.1.07

**Published:** 2022-01-28

**Authors:** O.V. Kurmyshkina, A.A. Bogdanova, P.I. Kovchur, A.I. Fetyukov, T.O. Volkova

**Affiliations:** Associate Professor, Department of Human and Animal Physiology, Pathophysiology, Histology, Medical Institute; Petrozavodsk State University, 33 Lenin Avenue, Petrozavodsk, 185910, Russia;; PhD Student, Laboratory of Ecological Biochemistry, Institute of Biology; Federal Research Center “Karelian Research Center of the Russian Academy of Sciences”, 1 Pushkinskaya St., Petrozavodsk, 185910, Russia; Professor, Department of Hospital Surgery, ENT Diseases, Ophthalmology, Dentistry, Oncology, Urology, Medical Institute; Petrozavodsk State University, 33 Lenin Avenue, Petrozavodsk, 185910, Russia;; Professor, Head of the Department of Hospital Surgery, ENT Diseases, Ophthalmology, Dentistry, Oncology, Urology, Medical Institute; Petrozavodsk State University, 33 Lenin Avenue, Petrozavodsk, 185910, Russia;; Professor, Head of the Department of Biomedical Chemistry, Immunology and Laboratory Diagnostics, Medical Institute; Petrozavodsk State University, 33 Lenin Avenue, Petrozavodsk, 185910, Russia;

**Keywords:** virus-associated cancer, human papillomavirus, RNA sequencing, transcriptome, 3D cell models, primary cancer cell culture, tumor microenvironment

## Abstract

The review summarizes findings from the studies based on the application of technologies for transcriptome analysis to modern cellular model systems of human papillomavirus-associated cancer (HPV) (cervical cancer, head and neck tumors). A diversity of three-dimensional cancer models, such as spheroids, organoids (organotypic cultures), explants, mouse xenografts, are addressed. Particular attention is paid to the use of patient-derived biomaterial for establishing short-term cultures of primary tumor cells, as well as generating multicomponent (heterocellular) systems that comprise, together with the tumor component, other elements of its microenvironment. A number of unique biological properties of HPV-induced neoplasia are discussed, which make generating cell models a unique task.

The novel findings in the field of molecular mechanisms of the onset and progression of HPV-associated cancer achieved by using RNA sequencing are presented for each variant of the model systems. These findings are considered in regard to applied aspects of their use, in terms of the opportunities for preclinical testing of new drugs, personalized diagnostics and selection of individual, most effective treatment regimens. The issues of drug resistance development, molecular-cellular heterogeneity, epigenetic reprogramming, and the role of the stromal microenvironment are reviewed. The paper accentuates the problems related to the limitations of the applicability of a particular model system. The areas with a significant lagging behind in omics research of virus-associated cancer in comparison with other types of oncological pathology and possible causes of this lag are noted. The future prospects for the development of model systems of HPV-associated tumors in the field of high-tech tissue engineering, in particular, the use of bioprinting and microfluidic biochips, are also outlined. The combination of these techniques with the methods of whole genome profiling will significantly increase the translational potential of the described model cell systems.

## Introduction

RNA sequencing (RNA-Seq) technique has contributed a lot to the development of transcriptomic and omics studies in general of malignant tumors of various histological types and etiology, including a group of virus-associated diseases, a significant proportion of the tumors being caused by human papillomavirus (HPV). The ability of RNA-Seq to compare the transcriptomes of surgical samples provides invaluable information on such phenomena and processes as genomic instability, cellular and molecular heterogeneity, antitumor and antiviral immune response, immune exhaustion, phenotypic plasticity, and multiple resistance. However, in many *in vitro* and *in vivo* studies, cellular models of human tumors are still indispensable, and then the purpose and design of research suggest the application of RNA-Seq to the model system. In turn, the widespread introduction of RNA-Seq and other options for high-throughput sequencing entails the development and modification of tumor models themselves [1].

Over the past 5–7 years, RNA-Seq has become a routine technique for the analysis of tumor cell lines growing in traditional two-dimensional (2D) culture, which are used in an experiment. At the same time, the understanding of the isolation of the processes in a model system, which is based on standard, genetically homogeneous cell lines, from the real physiological condition encourages the development of more complex systems. Most of the “classic” tumor lines obtained decades ago do not have a complete clinical annotation, and even their authenticity is often questioned today. Considering also the fact that any cell line is a clone of only one parental cell, which has turned out to be the most adapted one to growth in artificial culture, it becomes clear why many known tumor lines raise doubts today whether they really reflect the features of the original oncological pathology, are capable of reproducing all of its genetic and/or phenotypic diversity and can be used for translational research and solving problems of personalized medicine [1, 2].

**Figure 1:**
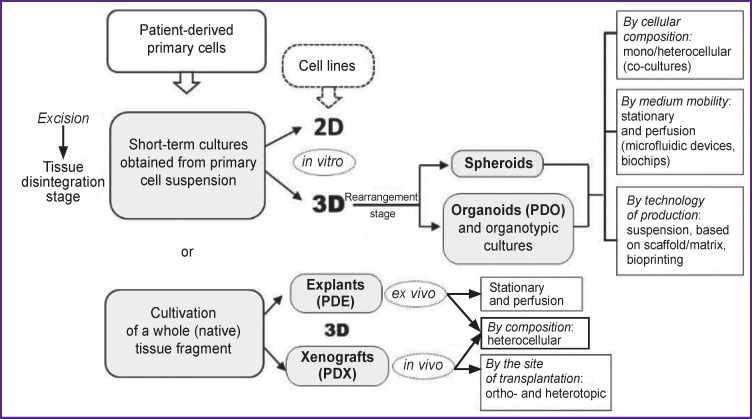
Diversity of cell models of human solid tumors

There are the following major trends in the development of modeling human solid tumors (Figure 1):

1. moving away from the monolayer to creating three-dimensional (3D) models;
2. refusing genetically homogeneous cell lines in favor of temporary cultures of patient-derived primary cells;
3. using heterocellular systems (co-cultures), including various components of the tumor microenvironment.

The most complex models available today are created as if at the intersection point of these trends and represent three-dimensional systems built from primary cells of various types.

Intensive implementation of tissue engineering technologies and the emergence of new cell models [3–7] can also be observed with regard to the localizations most often associated with viral carcinogenesis: first of all, it is cervical cancer (CC) and some head and neck cancers (HNC). As a result, we questioned whether and how widely the RNA-Seq technique is used in experiments with advanced model systems of HPV-associated (HPV(+)) cancer.

**The aim of the review** of the studies published on this issue is to understand how large is the unique body of information about HPV-dependent carcinogenesis, which was obtained using RNA-Seq of CC and HNC cell models, and at what points lagging in the development of this trend relative to other types of tumors of epithelial origin is observed.

Tumors caused by high-risk HPV are distinguished by their anatomical localization, occurring in the tonsils and their crypts, the pharyngeal wall, the root and base of the tongue, the soft palate, accounting for 20–30% of squamous cell carcinomas of the head and neck, as well as in the cervical region (its transition zone) and some other parts of the anogenital region. Histologically, the stratified squamous epithelial cells that line the surface of the mucous membranes are the source of HPV(+) cancer development. It should be noted that the very etiology of HPV-dependent cancer, together with its association with strictly defined epithelial sites, creates significant difficulties in obtaining tissue-like cell models [8]. The researchers underline that no adequate preclinical model systems for HPV(+) cancers are available yet [9]. Indeed, when analyzing the literature, we faced an obvious lack of experimental work on models of HPV-associated CC/HNC compared to other types of cancer, such as tumors of the female reproductive system (ovarian, endometrial cancer) or HPV-independent HNC. There are even fewer studies in which HPV-associated CC/HNC model systems are used to profile transcriptome changes using RNA-Seq.

**Figure 2:**
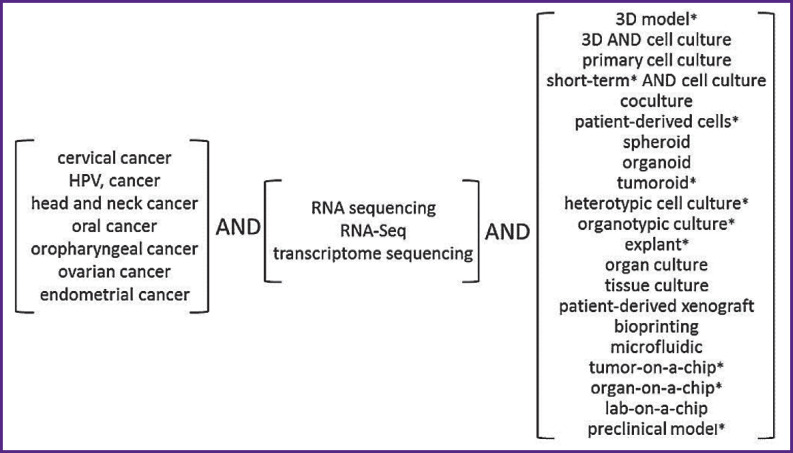
Scheme of used search filters: each search filter represented a combination of three terms, one from each group, in all possible combinations; * — not indexed in MeSH (https://www.ncbi.nlm.nih.gov/mesh)

The present review does not seek to conduct a rigorous systematic analysis of the published data; nevertheless, the methodology of literature selection includes some of its elements. The electronic search for publications was carried out using the PubMed database (https://www.ncbi.nlm.nih.gov/) in three groups of terms (Figure 2): the first group determined the type of cancer, the second one indicated transcriptome analysis, and the third one indicated the type of cell model. The search results included articles representing original studies with English-language full texts or abstracts, only if they clearly indicated the method of transcriptome analysis, the cell population used for modeling, and the source of its acquisition.

Based on the analysis, we identified a number of criteria for excluding publications from the review. First, we did not consider works in which RNA-Seq and cell model generating presented independent stages. Secondly, we did not take into account works in which the analysis of changes in the transcriptome of target cells was performed after their treatment with a conditioned medium of another culture or its separate fractions; although the design of such studies is often defined as indirect co-cultivation, it excludes, however, direct cell-to-cell interactions. For a similar reason, the review does not include studies in which individual cell populations are extracted from the material of the primary tumor and, after sorting, are immediately sent to obtain transcriptome libraries; in this case, the stage of creating a cell model and cultivation is absent. Thirdly, the review does not include studies using syngeneic mouse models of virus-associated carcinogenesis, or HPV-transgenic mouse lines. Fourthly, studies based on various genetic engineering manipulations with standard cell lines did not call our attention. When choosing a source of cells used to develop a model system, special attention was paid to primary cells derived from the patient biopsy (or surgical) material.

## Short-term (temporary) 2D primary cell cultures

### Monocultures of HPV(+) epithelial tumor cells.

As noted in Figure 1, obtaining a temporary culture of primary cells includes a stage of disintegrating the extracellular matrix in a sample of tumor or healthy tissue, after which the cells obtained in the form of a suspension can be sorted by phenotype and placed on a specially treated surface and in a culture medium to form a monolayer. In a study by Hayes et al. [10], on the example of a large panel of oral squamous cell carcinoma samples, it was shown, using exome sequencing, that it is short-term polyclonal cultures of primary cells at early (2–4^th^) passages that most accurately reproduce the mutation profile and, most importantly, the molecular heterogeneity of the donor tissue. However, establishing short-term cultures of primary cells, as well as new cell lines of HPV(+) cancer, is associated with rather great difficulties (low yield and rapid aging of the culture are among the most common problems), in consequence, for example, HPV(+) oropharyngeal cancer lines are significantly fewer than HPV(–) HNC lines [2, 11]. Obviously, it is caused by the rigid obligatory dependence of HPV(+) tumor cells on specific microenvironmental factors, which remain largely unknown and, therefore, cannot be reproduced *in vitro* [2]. A specific condition for cervical cancer that complicates the production of cell models is the source of its development, which can be keratinocytes of stratified squamous epithelium (ectocervix), single-layer glandular epithelium (endocervix), and the transformation zone separating them (metaplastic cells). Each of these populations may require specific cultivation protocols [12].

Another factor that potentially affects the viability of HPV(+) cells *in vitro* may be the presence of molecular subtypes that differ in the frequency and location of integration of the viral genome and, accordingly, in the frequency of mutations, expression of genes of epithelial, mesenchymal, and immune signatures. The total combination of the causes explains a small number of works on primary cultures of HPV(+) cancer, CC in particular, compared to other types of tumors [2]. Taking this fact into account, we should refer to a series of studies based on the use of normal primary epithelial cells (spontaneously or directionally immortalized) derived from various sources, including ecto- or endocervix, different parts of the oral cavity, pharynx and larynx, and also embryonic or neonatal tissues. The RNA-Seq application to the cultures of these cells transfected with HPV genes has contributed significantly to understanding the molecular basis of HPV biology in productive infection, and also significantly expanded the knowledge of the mechanisms of HPV-dependent carcinogenesis (Table 1).

**Table 1:** Results of RNA-Seq profiling of normal human epithelial cells when interacting with the human papillomavirus

References	Cells/lines	Core result
[13]	Spontaneously immortalized neonatal epidermal keratinocytes (NIKS)	The spectrum of signaling pathways reprogrammed in the “host” cell (keratinocyte) under the influence of HPV oncogenes *E6* and *E7* and depending on the life cycle of HPV (productive or unproductive, transforming infection) has been characterized
[14]	Spontaneously immortalized neonatal epidermal keratinocytes (NIKS)	
[15]	Primary normal epidermal keratinocytes (NHEKs)	
[16]	SV40 Т-antigen-immortalized embryonic kidney cells (HEK293T)	
[17]	hTERT-immortalized keratinocytes of the oral cavity (NOKs)	The function of non-oncogenic HPV proteins in the normal life cycle has been described
[18]	Primary neonatal epidermal keratinocytes (HEKa)	HPV-regulated profile of long non-coding RNAs (lncRNA transcriptome) has been decoded
[19]	Spontaneously immortalized keratinocyte line CIN612-9E derived from CIN1 (containing HPV 31 episomal form)	The mechanisms of HPV interaction with the interferon system of “host” cells have been revealed
[20]	HPV-immortalized normal ecto- (Ect1/E6E7) and endocervical (End1/E6E7) epitheliocyte line	The role of genomic rearrangements (by the example of chimeric gene formation) in transcriptome perturbations leading to transformation and carcinogenesis has been characterized
[21]	Primary cells of the normal squamous laryngeal epithelium (TVC-culture) and HPV immortalized TVC-HPVE6/E7 line	The contribution of physical and chemical environmental conditions to the increase in the risk of malignant transformation has been described

Here: CIN1 — cervical intraepithelial neoplasia grade 1.

Currently, DNA/RNA genomic sequencing (including whole exome sequencing, or WES) of primary monocultures of CC/HNC tumor cells is a compulsory type of analysis when obtaining a new tumor cell line for its complete molecular authentication and confirmation of histological affiliation [22]. RNA-Seq is used for the analysis of the profile of expressed mutations and the transcriptome-based identification, whether the cells of the resulting line belong to one of the molecular subtypes known for this type of cancer, as was demonstrated in the work by Fadlullah et al. [23] when obtaining a new panel of 16 HPV(+) and HPV(–) squamous cell carcinoma lines from different parts of the oral cavity. Nevertheless, in this type of work sequencing is used mainly as an auxiliary method — to outline a “portrait” of a new line, whereas, in a number of other studies, WES and RNA-Seq of primary cultures address fundamental issues. Unfortunately, this area has not actually been developed in relation to cervical cancer: one study can be cited [24], in which genomic profiling was performed on the material of 15 HPV(+) primary cell lines from patients with cervical cancer, but only at the exome level, to establish the mutation profile.

In relation to HNC, there are a few more publications in this area. For example, the study by Pirotte et al. [11] is aimed at elucidating the causes underlying the differences in the clinical picture of HPV(+) and HPV(–) HNCs, namely, sensitivity to radiotherapy and a number of targeted drugs (HPV(+) HNCs are known to be mainly susceptible to radiation and have a better prognosis). The authors supposed the HPV status-associated differences in the state of the DNA repair system to determine differences in sensitivity to PARP inhibitors. To test the hypothesis, a panel of 8 HPV(+) and HPV(–) HNC lines was used, two of which were obtained by culturing explant specimens and represented HPV(+) tonsillar cancer. The result of RNA-Seq analysis was quite unexpected: no significant differences were found between HPV(+) and HPV(–) lines in the expression of genes responsible for the repair of single-/double-strand breaks, nitrogenous bases, components of the APOBEC- and p53-dependent response, and the PARP1 factor itself. With regard to PARP inhibitor olaparib, cells of different lines demonstrated differences in sensitivity, but they were not associated with either HPV status or the level of PARP1 expression, which proves the need to search for additional markers to predict the sensitivity or resistance of HNC to PARP inhibitors. Pirotte et al. [11] note that the differences in the clinical and pathological manifestations of HPV(+) and HPV(–) HNC are based most likely on the features of the immune response, rather than the DNA repair system or p53-dependent apoptosis.

### Primary cell cultures as models for the development of drug resistance.

Solving the issue of the development of tumor resistance, studies involving cultures of patient-derived primary tumor cells begin to play an important role due to the opportunity to establish the whole variety of natural mechanisms that lead to the same result at the clinical level, namely, drug resistance, but require different approaches to combat it. Nevertheless, until now, the investigation of the causes of CC/HCC resistance has been carried out, as a rule, on standard tumor lines. It should be mentioned that even in this case, the production of resistant clones includes a stage of cell evolution under the pressure of increasing concentrations of a chemical agent and, therefore, is probabilistic in nature, and cell populations with a new resistant phenotype will be genetically heterogeneous. The RNA-Seq analysis of resistant sublines is used to outline the range of possible causes of the resistance to cytotoxic or targeted therapy at the transcriptome level, as well as identify phenotypically significant mutations.

Currently, the adaptation mechanisms that are realized over time through step-by-step transcriptomic reprogramming and form the basis for epigenetic plasticity of tumor cells are of particular interest. In practical terms, such *in vitro* experiments allow the discovery of new potential biomarkers of resistance, which can then be analyzed retrospectively on a large number of transcriptome profiles of tumor samples stored in the TCGA dataset (The Cancer Genome Atlas: https://portal.gdc.cancer.gov), and thus harvest a certain amount of clinically relevant information (Table 2 contains a selection of studies on this issue).

**Table 2:** Results of RNA-Seq profiling of resistant phenotypes obtained by targeted selection of “parental” cells of standard cancer lines

References	Cells/lines	(Active their combination) ingredient	Core result
[25]	CC (HeLa/DDP)	Cisplatin	Key role of the PI3K/AKT-dependent pathway
[26]	HNC (CAL27 and SCC9)	Cisplatin + 5-fluorouracil	The leading role of the Hedgehog pathway in the activation of the ABC transporter system and the development of multidrug resistance
[27]	HNC (SCC25)	Cetuximab	The importance of delayed epigenetic stabilization
[28]	HNC (SCC1, SCC6, SCC25)	Cetuximab	of the EGFR-inhibitor-resistant phenotype
[29]	HNC (CAL33 and CAL27)	Proton radiation	The role of VEGF-C-dependent mechanisms in resistance development
[30]	HNC (CAL27)	Anoikis	The leading role of VEGF-dependent mechanisms

Although tumor cell lines provide virtually unlimited possibilities for varying experimental conditions, the development of a stable phenotype can also be modeled in the culture of primary tumor cells. For example, in a study by Low et al. [31], they performed a comparative RNA-Seq analysis of three cell lines obtained from the patients with HNC who were found to be sensitive to gefitinib (a low molecular weight tyrosine kinase inhibitor of the 1^st^ generation), and isogenic resistant lines. The resistance acquired *in vitro* turned out to be not at all associated with genomic disorders, in particular, changes in the structure of the *EGFR* gene, but develops at the phenotypic level through large-scale changes in the transcriptome profile, particularly, through activation of the expression of epithelial-mesenchymal transition (EMT) and interferon-dependent response genes. This entails resistance to 2^nd^ and 3^rd^ generation EGFR inhibitors and may be one of the causes for the low efficacy of these drugs against EGFR(+) HNC in clinical trials.

In general, the problem of the development of drug resistance is associated with the problem of intratumoral heterogeneity and clonal evolution. The key to the survival of tumor cells under the pressure of a selective factor is their phenotypic diversity, which is supposed to be achieved by implementing two strategies [27, 31, 32]. The first one is based on the principle of “Darwinian evolution” and implies the preexistence of genetically heterogeneous cell populations in the initial tumor, among which the ones most adapted to this factor survive (get a proliferative advantage). The second, alternative, strategy is based on the principle of adaptive evolution, i.e., the expansion of phenotypic diversity in an initially genetically homogeneous population, induced by the selective pressure of the environment [32]. In the first case, the mechanism of generation of the HPV(+) CC/HNC clonal diversity is known — it is a consequence of the impaired functioning of oncosuppressor genes (for example, HPV oncogenes *E6* and *E7* inactivate the p53 and pRb proteins of the “host” cells, as a result of which these cells can acquire mutator phenotype). The mechanism proposed for the second strategy is the global reprogramming of the cell transcriptome within the tumor [32]. This method is associated with new ideas about epigenetic/transcriptional plasticity, i.e. the ability of tumor cells in a metastable state to move into *de novo* phenotypic states and fix them without making genetic changes (“phenotypic switching”). Obviously, both of these strategies are implemented in the course of natural tumor progression, but only the analysis of single cells in the model system allows evidence of their joint contribution to the resistance at the level of the whole tumor.

This hypothesis was tested in the study by Sharma et al. [32], which was performed using single-cell transcriptome sequencing technology (single-cell RNA-Seq, scRNA-Seq) on the material of primary cultures obtained from therapeutically “naive” samples of oral squamous cell carcinoma. In the initially heterogeneous culture, the researchers managed to detect a rare population of cells with a transcriptome profile reflecting preexisting cisplatin resistance, and then observe a gradual expansion of this population in the presence of cisplatin. At the same time, stress-induced cell transdifferentiation associated with changes in the expression of a number of epithelial/mesenchymal markers and the emergence of a new cisplatin-resistant phenotype was recorded in the initially phenotypically homogeneous culture. The authors also traced the fixation of the developed resistance throughout at least 20 passages of cultivation after the removal of cisplatin, which indicated the involvement of epigenetic regulatory mechanisms. Transition into a new state was caused by a “switch” between the differentiation programs triggered by the master transcriptional regulators SOX2 and SOX9. Today, both of these transcription factors are believed to be stem cell markers, and, according to the results of the study by Sharma et al. [32], SOX2 has also turned out to be a marker of epigenetic plasticity of tumor cells. The authors confirmed the clinical significance of the regularities identified in the model system by registering similar changes in the foci of secondary tumor growth in the same patients after a course of cisplatin therapy. These findings lead to believe that different strategies for tumor cell survival will require different approaches to diagnosis and treatment.

### Cultures of primary tumor cells as a test system for response to therapy.

When considering the practical aspect of using primary tumor cell cultures, it should be noted that models based on them help in solving several problems. Firstly, they are used in applied pharmacogenomic research to establish the determinants of sensitivity to drugs, search for new molecular targets that would allow to bypass the developed drug resistance, test new drugs or their new combinations. Secondly, primary cultures are used as preclinical cell models to assess individual drug response and predict treatment efficacy. The role of RNA-Seq in the studies in these areas is increasing, since DNA sequencing and obtaining a cancer mutanome cannot always indicate the likely effect of any targeted drug. The relationship between mutations and the functioning of protein targets of such drugs is often indirect and difficult to trace, particularly in the case of HPV(+) cancer with its large variety of low-frequency mutations. In addition, not all functional groups of genes and their mutations are amenable to targeting.

This fact is illustrated in the study by Zammataro et al. [24] where 15 primary cell lines of HPV(+) cervical cancer were obtained and their exome was sequenced at early passages. Further, based on the profile of genomic aberrations for each line, a targeted drug was selected and tested. Most of the identified mutations affected the ERBB2/PI3K/AKT/mTOR pathway; however, for specimens with the mutant *PI3KCA* gene, the selective PI3K inhibitor copanlisib showed low activity in primary cell cultures. Hence, although *PI3KCA* is the most frequently mutated gene in squamous CC (27.1% according to TCGA [33]), it itself is a weak driver of oncogenesis, and its blocking causes the activation of compensatory mechanisms [24]. The researchers [24] emphasize the importance of profiling the consequences of genetic aberrations at the transcriptomic and other levels of regulation in order to establish relationships with the therapeutic effect. This was also demonstrated by Xu et al. [34], who used a culture of primary cells of cisplatin-resistant squamous cell carcinoma of the oral cavity at early passages for WES and RNA-Seq. The analysis of the cell genomic profile, regardless of the high mutation load, did not allow making a univocal therapeutic choice: the majority of genetic events (point mutations, amplifications/deletions, rearrangements) and even overexpressed genes turned out to be phenotypically neutral, and only the functional screening by the small interfering RNA libraries was helpful to narrow the search up to a countable number of targets potentially lethal to tumor cells. A similar study design was presented by Sa et al. [35], who obtained a collection of more than 100 short-term cultures of primary tumor cells from patients with CC, ovarian and endometrial cancer. The cell cultures were treated with a panel of 37 targeted drugs, then their response was compared with the results of exome and transcriptome sequencing of cells in the original tumor. Thus, RNA-Seq of model cell systems is becoming a necessary component of research aimed at developing a targeted approach to the administration of highly selective anticancer drugs.

### Primary cell cultures of the tumor stroma.

Cancer-associated fibroblasts (CAFs) are the main non-immune component of the tumor microenvironment. Under the impact of tumor cells, normal fibroblasts can undergo phenotypic reprogramming, so that they are even said to undergo malignant transformation, implying the acquisition of a stable CAF phenotype. In turn, CAFs continue to transform the tumor microenvironment increasing its aggressive properties. Today, there is an understanding that CAFs should become a target of complex antitumor therapy, but in the case of virus-associated CC/HNC, this issue has not been sufficiently developed. Back in 2016, Kumar et al. [36] described the transcriptome of primary CAFs isolated from CC samples of early and late stages and transferred to culture, though, the method of analysis was chosen to be hybridization microarrays. Similarly, in 2018, the primary CAF transcriptome from oral cancer was analyzed [37], the authors being able to identify the phenotypic heterogeneity of CAFs, which can be considered as a component of intratumoral heterogeneity. However, in contrast to the papers on cervical cancer, publications on HNC have begun to appear, presenting transcriptomic studies of temporary CAF cultures performed with the use of RNA-Seq. For example, Takahashi et al. [38] obtained CAF cultures and normal fibroblasts from HNC patients and performed a comparative transcriptome analysis at early passages. They found 13 differentially expressed genes associated with immunosuppression. In the study [39], the RNA-Seq was used to compare CAFs and normal fibroblasts obtained in a short-term culture from HNC patients according to the expression profiles of long non-coding RNA (lncRNA) and their mRNA targets in order to identify signatures associated with fibroblast transdifferentiation in CAFs. Among the differentially expressed genes, CAF-specific activation of intergenic lncRNA (LOC400221) was found and its association with the pro-inflammatory cytokine IL-33 expression in the tumor stroma was established, which confirms the concept of CAF participation (and, possibly, the leading role) in maintaining the inflammatory microenvironment promoting tumor progression.

Another important component of the tumor stroma is vascular endothelial cells, which can actively participate in inflammatory reactions. In a recent work by Lopatina et al. [40], tumor-associated endothelial cells were isolated from stage IV HNC and transferred to a temporary culture, after which the RNA content of their extracellular vesicles was analyzed by RNA-Seq. The vesicles were enriched with transcripts responsible for the regulation of inflammation, T/B cell activation/immunosuppression, and controlling TGF-β- and interleukin-dependent signaling pathways. Moreover, incubation of primary mesenchymal progenitor cells in the presence of endothelial vesicles induced transcriptome changes in them consistent with a pro-inflammatory and immunosuppressive phenotype. Therefore, not only tumor cells themselves but also endothelial cells can participate in the spread of pro-oncogenic signals through the secretion of vesicles and stimulate the reprogramming of the immune microenvironment.

### Heterocellular cultures (co-cultures).

Modelling of the direct interaction of tumor cells with their microenvironment is possible through co-cultivation. A combination of primary cells and cell lines is often used to standardize the source of exposure, and the changes in the cells, which are the research object, are recorded using various high-throughput methods. Thus, in a recent study [41], the RNA-Seq analysis of changes in the transcriptome of peripheral blood neutrophils from healthy donors was performed after their short-term co-cultivation with HeLa cells. Some of the neutrophils were pretreated with resolvin D1 (an anti-inflammatory lipid regulator of local action). The data obtained indicated the participation of neutrophils, “instructed” by HPV(+) tumor cells, in pathological inflammatory responses, and the treatment with an anti-inflammatory agent, on the contrary, contributed to the reprogramming of the neutrophil phenotype to an antitumor one.

A similar study design was presented in the work [42], where RNA-Seq was used to evaluate changes in blood mononuclear fraction after co-cultivation with HNC-line cells.

A co-culture model is also used to reproduce the relationship of tumor cells with the primary mesenchymal stem cells mentioned above. Here, it is important to note really mutual influence of these two types of cells: on the one hand, tumor cells are able to direct the differentiation of mesenchymal cells, and they, in turn, can enhance or weaken the aggressive properties of tumor cells. This issue is dealt with by Liu et al. [43], who analyzed the transcriptome changes in HNC-line cells after their co-cultivation with mesenchymal cells derived from bone marrow. The RNA-Seq analysis showed that such tumor cells have a higher level of EMT gene expression and drug resistance. A similar study was carried out for cervical cancer cells (HeLa line) and primary mesenchymal stem cells isolated from adipose tissue; the up-regulation of EMT signaling pathways, angiogenesis, and inflammation in HeLa cells under the action of mesenchymal cells was confirmed [44].

## 3D spheroids (oncospheres, tumorospheres)

Spheroid cultures are three-dimensional aggregates into which cells spontaneously assemble under required conditions. If spheroids are enriched with the properties of tumor stem cells, they can be called tumorspheres; in this case, the spheroid is a clone of the parent cell [2]. It is believed that a single spheroid roughly corresponds to a tumor microdomain (a microregion of tumor tissue enclosed between the branches of the capillary network), so this model better reproduces physicochemical gradients and differences in proliferation/apoptosis/necrosis rates within a single microsite. As the authors of systematized reviews note [45–47], the use of spheroid cultures and other 3D models in HNC/CC studies is in the initial stage of development (mainly in the stage of optimizing experimental protocols), while the area of cell spheroid application is limited mainly to routine types of analysis in which the ability of cells to spheroidogenesis is considered only as an indicator of the degree of expression of the somatic stem cell traits. If we turn to works that use a spheroid culture precisely as a model for recreating a certain cell state, which is then analyzed using RNA-Seq, then we can see that spheroids for cervical cancer (cervospheres) were derived from standard tumor lines (HeLa, SiHa, CaSki), but not from patient-derived primary cells. The situation is similar for other HPV-dependent tumors. The features of the HPV life cycle, a high dependence of proliferation/differentiation/survival of normal and transformed keratinocytes on environmental factors and the composition of the extracellular matrix, which together predetermine serious methodological difficulties in obtaining viable primary tumor spheroids, offer a likely explanation for that [45].

In the study by Yang et al. [48], the RNA-Seq analysis was applied to spheroid cultures of HPV 16(+) cervical cancer cell lines (SiHa and CaSki) to elucidate the role of the *E7* oncogene in maintaining the stem properties of cells and the mechanism of this effect. Another study [49], using miRNA-Seq, demonstrated that the composition of transcripts in exocytotic vesicles of HeLa cells growing in the form of 3D spheroids is closer to the miRNA composition of vesicles isolated from the patient-derived plasma, compared with HeLa cells growing in a monolayer (2D). The few works in which cervospheres or orospheres were obtained from patient primary cells mainly study the properties of tumor stem cells and the role of HPV in their maintenance [5, 50–53]. In general, these works indicate a higher physiological and clinical relevance of the spheroid model and, accordingly, greater prospects for it as a tool for personalized medicine, but the RNA-Seq method has not yet been used in them.

## 3D organoids (microtissues, miniatures of organs)

Organoids are self-organizing and self-maintaining *in vitro* multicellular structures that reproduce the histology and microphysiology of a particular organ or part of it. As a rule, they consist of cells belonging to various differons, being “mini-replicas” of any tissue. Epithelial organoids can be derived not only from malignantly transformed cells but also from normal epitheliocytes so as to reconstruct the earliest events of carcinogenesis. Generation of a healthy tissue organoid is based on proliferation of the adult somatic stem/progenitor cells in the donor tissue and their transfer to the environment that reproduces a so-called niche. The correct selection of niche microenvironment factors provides realization of the internal, autonomous ability of the cells of an organoid culture to spatial self-organization. The common opinion suggests that organoids or organotypic cultures isolated from the cellular material of patients (patient-derived organoids, PDO) are the most optimal compromise between *in vitro* and *in vivo* systems, combining the experimental flexibility of *in vitro* culture and the complexity of the *in vivo* model [1], and, therefore, are evaluated as the most promising preclinical 3D model systems.

### Organoid cultures of normal and tumor tissue of the mucous membrane of the cervix and oral cavity.

Different authors note it to be challenging to obtain an organoid culture of virus-associated tumors, especially HPV(+) cervical cancer, therefore, there is a significant lagging behind compared to other types of cancer in this area of research. Obviously, not all required “niche” factors specific to the cervical epithelium are known [54]. Lõhmussaar et al. [55] aimed their study at the development of an experimental platform for cervical cancer and normal ecto-/endocervical epithelium based on organoid culture. In this work, RNA-Seq was used to analyze the transcriptomic profiles of the specimens of healthy epithelium, cervical cancer, and organoids grown from them. The obtained findings have confirmed that the organoid culture maintains the molecular characteristics of the original tissue for a sufficiently long time; ecto- and endocervical organoids and tumoroids indeed have different gene expression profiles; tumoroids retain the viral sequences of various HPV subtypes and provide their expression.

Quite recently, a study by Chumduri et al. [54] was also published in which organoids of normal endo- and ectocervix were obtained from the patient-derived biomaterial in order to decode the mechanisms for maintaining cellular homeostasis of the transition zone between the stratified squamous and single-layer glandular epithelium. Moreover, the authors were interested in the causes and mechanisms of remodeling the “niche” of the transitional zone in the process of squamous metaplasia, which is often a prerequisite for the development of CC. The ectocervical organoids had a multilayered architecture with a typical layer differentiation for squamous epithelium, while the endocervical cells gave rise to hollow organoids with a single-layer lining of prismatic/columnar cells. The transcriptome analysis of organoids was performed using cDNA microarrays rather than RNA-Seq, but, nevertheless, the created model gave rise to a number of clinically significant conclusions. It has been established that the epithelium of the ecto- and endocervix originates from different lines of stem cells, the stem cells of the both differentiation lines being distributed in a mosaic manner. The choice of the line which will start proliferation and differentiation in this particular area of the cervix is determined by the microenvironment of the underlying stroma, namely, the “niche” factor gradients created by it. Accordingly, the primary cause of remodeling of the transformation zone and metaplasia is the reorganization of the stromal microenvironment. Such niche factors specific to ecto- and endocervical cells have been found. In particular, the WNT3A factor has turned out to be a repressor of endocervical differentiation. The comparative analysis of transcriptomes of ecto- and endocervical organoids at different cultivation periods revealed differences in the regulation of the WNT-dependent program, in the expression of members of the Notch signaling pathway, and in the pattern of cytokeratins (KRT). The overlay of transcriptome profiles of organoids on the transcriptome profiles of CC samples from the TCGA dataset indicated the most likely source of squamous cell carcinoma or cervical adenocarcinoma: in the first case, this is the KRT5(+) stem cell lineage, in the second one, this is the KRT7(+)KRT8(+) lineage. The authors note a need to search for diagnostic markers and approaches to the CC treatment that are specific to these cell lineage.

Maru et al. [56] derived organoids directly from the cellular material of the cervical transformation zone. The organoids had a unique morphological structure and shape but generally corresponded to the histological organization of the original tissue site. The transcriptome analysis of the organoids, performed by means of cDNA microarrays, showed the preservation of the expression of metaplastic epithelium markers and, consequently, they can be used as a relevant model for the experimental induction of carcinogenesis. It is curious that a little earlier, a paper describing the generating of an organoid culture from a patient with an exceptionally rare type of CC, clear cell adenocarcinoma, was published [57], but the genomic profiling of organoids was carried out only to establish the correspondence of their mutational profile to the original tumor tissue.

The organoid trend for HNC is also being actively developed, but the low efficiency of methods for generating PDO limits so far the widespread use of RNA-Seq and other genomic types of analysis [58, 59]. In general, the current studies are devoted to improving the protocols for their generating from biopsy samples of healthy and tumor tissues and working off experimental exposure [60–64], while it is noted that genome-wide/transcriptome studies are urgently needed to substantiate the predictor potential of HNC organoids [58]. In one of these studies [65], RNA-Seq was applied to PDO isolated from the samples of normal oral mucosa and tumors developing from various anatomical regions of the oral cavity and its derivatives (floor of the mouth, tongue, gums, pharynx, larynx, salivary glands as well as nasal cavity and neck), however, the transcriptome analysis in that case solved the problem of exclusively characterizing the organoid model itself, namely, confirming the differences in gene expression profiles between normal and tumor organoids. To study the processes preceding the development of HPV-associated CC/HNC, raft cultures are also used as organoids relevant to stratified epithelium [66].

### Organotypic raft cultures.

Raft cultures reproduce the vertical layer differentiation of stratified squamous epithelium, on which the HPV life cycle is highly dependent. If the cells of such a culture are transfected with HPV genes or infected with HPV quasivirions, then it is possible to model a latent/productive/transforming infection and associated changes in keratinocytes, morphologically corresponding to dysplasia (intraepithelial neoplasia) of varying severity. Since there are very few stable cell lines derived from precancerous pathologies (for example, there are only two lines of cervical intraepithelial neoplasia cells for the cervix), organotypic cultures are built, as a rule, from primary human keratinocytes (neonatal or adult ones, derived from various anatomical sites) placed on a feeder layer of fibroblasts. The use of RNA-Seq in such a model system provides the assessment of the HPV and its individual protein effect on the cell transcriptome [67–69] to characterize the signaling pathways, the deregulation of which leads to carcinogenesis, and to correlate the observed changes with morphological abnormalities in the raft [70].

Unfortunately, not all the localizations are the source of keratinocytes which accurately reproduce the differentiation programs of the epithelium of the cervix or oral mucosa [66]. The transcriptome analysis of raft cultures grown from keratinocytes of various origins (cervix, gums, tonsils, etc.) showed that the cellular response to HPV infection in them differs considerably, as well as the levels of expression of immune response genes and epidermal differentiation are different (which, by the way, may underlie a specific susceptibility of cervical keratinocytes to HPV-induced carcinogenesis) [71, 72]. Thus, organotypic raft cultures from primary epithelial cells of appropriate localization better suit for modeling viral infection and its consequences [58].

The procedure of deriving organotypic cultures from primary cells of normal epithelium and cervical neoplasia using human cervical stromal cells is described in the papers [73] and [74], but examples of using RNA-Seq are not yet available. Quite recently, the detailed protocols have been published for different approaches to generating HPV(+) and HPV(–) organotypic 3D models from primary epithelial cells of the oral cavity and cervix with examples of their application, including omics studies [45], which suggests greater use of next-generation sequencing technologies.

An organotypic raft culture can be generated not only for stratified normal or dysplastically altered epithelium but also for early, intraepithelial cancer; in this case, it can serve as a model of early invasion. This possibility was demonstrated in two works on cervical cancer [75] and [46], while the study by de Gregorio et al. [46] paid particular attention to generating a 3D stroma model, consisting of primary tumor-associated cervical fibroblasts and the extracellular matrix also produced by them. The authors point out that the connective tissue base in such a culture is not just a substrate, but an equal participant in crosstalk between tumor and stromal cells, and it is the authentic stroma that properly reproduces the signals of the microenvironment necessary for cervical cancer cells to undergo EMT and activate invasion. In both the works, the cells of standard CC tumor lines (C33A and SiHa) were used as a tumor component of the raft model, and their analysis was limited to the determination of individual markers without the use of RNA-Seq.

Along with many advantages, an organoid culture as a 3D cell model has obviously a number of disadvantages. Organoids do not always allow to reproduce all the components of the morphological structure of the original tissue [57]. A common disadvantage of organoids is a gradual loss of certain tumor clones, the proliferation of fibroblasts, and the absence of blood vessels. Unfortunately, the rearrangement of the immune microenvironment in the organoid culture of HPV(+) CC/HNC also remains an unsolved problem [1]. Some of these limitations are aimed to be eliminated by explant culture.

## Explants

An explant culture is a microfragment (1–2 mm^3^) of native tissue cultivated *ex vivo*. Thus, by definition, it is an organotypic 3D model obtained from patient cells (patient-derived explant, PDE). A valuable advantage of the explant model is that it preserves tumor cells in their original microenvironment, including vessels, stroma, immune infiltrate, and the absence of enzymatic treatment keeps all intercellular interactions intact [2]. In addition, explants of normal and tumor tissues provide possibilities to conduct comparative transcriptomic studies, including those after their treatment with chemotherapeutic agents, for a preliminary assessment of an individual response to therapy. Due to the preservation of the immune microenvironment, RNA-Seq in this case allows to reveal, practically *in situ*, new mechanisms of action of immunotherapeutic antitumor agents. The methods for obtaining explants and culturing them in the presence of chemical agents have been previously described in relation to HPV-associated cancer and precursor intraepithelial neoplasia lesions (for example, [76, 77]). Moreover, HPV(+) explant culture has shown the ability to reproduce the features of the immune response to drug treatment, but as a model, it has not yet been widely spread in genomic/transcriptome studies of CC/HNC, unlike other types of cancer.

Despite the fact that the explant culture is considered along with organoids as a very promising experimental cell system, it has a number of limitations: the use of an explant implies very short cultivation periods (several days); the absence of natural tissue perfusion and the gradual expansion of necrosis inevitably cause changes in the transcriptome profile. This problem can be solved partially with an *in vivo* model.

## Patient-derived xenografts

Xenografts (“avatars”, patient-derived xenografts, PDX) are tumors obtained by transplanting the patient-derived cellular material into the body of a laboratory animal (usually, mice) with genetic defects in the immune system that prevent transplant rejection. In studies on cervical cancer, the use of PDX as an experimental model is very uncommon, since their obtaining has proved to be too challenging to perform genomic analysis [78]. It is also obvious that the viral nature of CC demands an orthotopic microenvironment for a tumor graft in order to avoid significant distortions in the profile of tumor cell expression.

Orthotopic PDX models of cervical cancer have actually been developed [50, 79]. Besides, a method was described for obtaining PDX cultures from intraepithelial neoplasia of the cervix by transplanting to mice under the renal capsule [80]. In these studies, the main task was to confirm the preservation of the original histological organization, which did not involve the use of genomic profiling, molecular screening, or similar methods. In the work by Rofstad et al. [81], the possibility of reproducing such properties as the individual density of the intratumoral lymphatic microvasculature of patients, invasiveness, and metastatic activity in the PDX culture of CC was shown at the morphological level. On the other hand, some authors express doubts about the stability of the morphological and molecular properties of cervical cancer (including HPV status) in the form of PDX and believe that with serially transplanted PDX, significant deviations from the properties of the patient-derived tumor may be observed [82]. Doubts about the relevance of PDX models of CC could be resolved by comparative genomic/transcriptome studies. However, so far we can talk about the use of RNA-Seq only for tumor xenografts grown from the cells of standard human lines, such as HeLa, transplanted into mice. As a rule, the design of such studies is based on experimental treatment of tumor cells in an *in vivo* environment and analysis of their response at the transcriptomic level [83, 84].

Due to the different nature of HNC carcinogenesis, it is possible to compare the efficiency of obtaining PDX for HPV(+) and HPV(–) tumors. The yield of PDX cultures of HPV(+) HNC has turned out to be significantly lower than that of HPV(–). Thus, we can speak about the low survival rate of HPV-dependent tumors (CC, HNC) when transplanted into a foreign environment as a general pattern, so the problem of adequate *in vivo/ex vivo* models for them is particularly relevant [85–88]. There is an ongoing search for the causes underlying the differences in the properties of HPV(–) and HPV(+) HNC. For example, one of the causes was previously associated with differences in the phenotype of tumor stem cells, but Keysar et al. [89] demonstrated, using RNA-Seq analysis of PDX models enriched with the ALDH+CD44high phenotype, that HPV(–) and HPV(+) HNC stem cells have a similar profile of signaling pathway activation. In any case, for PDX models of HPV(+) HNC using exome sequencing, evidence was obtained for the preservation of a unique pattern of genetic aberrations for each patient, including potentially targetable mutations, which suggests the possibility of their use as preclinical models [9].

The study by Lilja-Fischer et al. [90] gives an example of the application of the transcriptome RNA-Seq analysis in a heterotopic PDX culture of HPV(+) HNC. It is interesting to note that despite the reproduction of morphological characteristics, HPV carriage, and the profile of key mutations, xenografts had a number of differences from the original tumor samples in terms of gene expression profile. The genes responsible for the immune response and response to hypoxia and mesenchymal genes showed a reduced level of expression in PDX. The authors attribute these differences to the unnatural microenvironment of PDX: the weakly vascularized subcutaneous area of transplantation alters the activity of genes that control the response to hypoxia, and the immunodeficient status of the recipient mouse distorts the mechanisms of tumor resistance to the host immune system.

Nevertheless, attempts are being made to optimize the study design so that HNC PDXs meet the requirements of a predictor model and aid in studying the mechanisms of drug resistance *in vivo.* In 2017, the results of transcriptomic profiling (on cDNA microarrays) of 28 PDXs of squamous HNC were published, after which the experimental animals were given cytotoxic or targeted drugs. The drug response was compared with a transcriptome profile to extract a predictor expression signature [91]. And more recently, the work by Yegodayev et al. [92] illustrated the potential of RNA-Seq in the *in vivo* analysis of the effect of targeted therapy in a HNC PDX culture: PDX mice were injected with cetuximab (an anti-EGFR monoclonal antibody drug), after which they were divided into two groups, those responding to therapy by reducing xenograft size and those treatment-resistant. After comparing the transcriptomes in these two groups with the PDX control group, it was found that the response to cetuximab in the sensitive group was due to changes in the stromal (mouse) component, and tumor progression under cetuximab was due to CAF activity. In general, an important advantage of xenograft models is their ability to distinguish the contribution of tumor cells themselves and the stromal component (based on mouse reads) to the phenomenon under study using next-generation sequencing technologies [93].

In addition to the involvement of stromal cells, another cause of resistance to targeted HNC drugs *in vivo* may be the activation of compensatory mechanisms. In this case, PDX studies using RNA-Seq prove to be useful in search of therapeutic ways to “bypass” this cause. For example, Bhatia et al. [94] analyzed in their work the contribution of the EphB4/ephrin-B2 signaling pathway to the decrease in the effect of EGFR inhibitors, cytotoxic and radiation therapy on the oral cancer PDX material. The authors deliberately derived PDX models of HPV(+) and HPV(–) HNC from patients with initially high expression levels of EphB4, ephrin-B2, and EGFR. The mice were then treated with medications (cetuximab, cisplatin) with or without the addition of the EphB4/ephrin-B2 ligand-receptor interaction inhibitor. After obtaining differences in response to different combinations of medications and comparing the transcriptome profiles of PDX tumors, differences in the expression of downstream targets of the EphB4/ephrin-B2 pathway were found.

With regard to HNC, a new format for clinical trials of anticancer drugs is also being developed, based on the parallel observation of the response of the patient and “their” mouse. At the same time, the combination of genomic and transcriptomic profiling methods contributes to a deeper understanding of the causes for greater effectiveness of certain combinations of drugs and the mechanisms of resistance development. The outcomes of the trial in this format for the pan-PI3K inhibitor buparlisib and its combination with cetuximab have been recently presented by Kim et al. [95]. According to RNA-Seq data, the higher efficiency of the combination of two drugs in the PDX model is due to a stronger activation of the apoptosis-related gene expression and negative cell cycle regulators. A similar platform for coupled preclinical and clinical trials with verification of observation by genomic analysis methods is described by Campbell et al. [96] using the PDX model of oral squamous cell carcinoma and the MEK1/2 inhibitor trametinib as an example.

The absolute limitation of the PDX model is the absence of the immune system of the recipient organism. Although this fact sometimes facilitates “revealing” specific mechanisms of innate response of tumor cells, usually masked by immune cells, tumor PDXs are not suitable for research in the field of immuno-oncology and testing immunotherapeutic antitumor agents. PDX models need to be improved to realize this possibility, transition from the use of immunodeficient mouse lines to the use of partially or fully humanized lines capable of developing an immune response, including an inflammatory response, is necessary. This is particularly important for HPV-induced tumors that arise, progress, and respond to therapy in permanent interaction with the host immune system [97].

## Bioprinting and microfluidic devices (tumor-on-a-chip)

### Bioprinting.

*In vivo* laboratory animal models are expected to be replaced in the near future by multicomponent 3D tumor cultures printed from patient-derived cellular material [98]. Particularly promising are the possibilities of creating specimens of vascularized tumors that reproduce spatiotemporal chemical gradients of bioactive factors and contain immune infiltrate and stroma so as to model the dissemination of tumor cells through invasion/intravasation and stimulation of angiogenesis, choose an individual treatment regimen and predict more accurately an individual response to therapy under near-physiological conditions [99, 100].

Studies using transcriptomic analysis and 3D printed tumor cell structures have emerged quite recently, in fact within the last two years. The technology for creating 3D bioprinted models from patient-derived primary epithelial tumor cells is still at the initial stage of development [101]. Bioprinting of cervical cancer is limited by optimization of the printing methods themselves using the standard HeLa line as “bioink” [102, 103]. Vascularized HNC model printing also remains in the phase of reviewing and selecting conditions [104]. Taking into consideration that omics studies using 3D printed modeling systems are rapidly developing for many types of non-viral and non-epithelial tumors, the same progress is expected for HPV-dependent neoplasias.

***Microfluidic devices (chips),*** or microphysiological systems provide cultivation of any of the 2D/3D model systems described above under controlled conditions that mimic natural blood flow. Along with bioprinting, “tumors-on-a-chip” or “organs-on-a-chip” are the cutting-edge high-tech types of cell model systems. As for epithelial tumors and their corresponding normal tissues, only a few studies have been found so far that combine this culture technology and genomic methods of analysis. For squamous cell carcinoma of the oral cavity, there is a case of the use of a microfluidic device, which aided in modelling the process of tumor invasion and then isolation of a microRNA fraction from the cells of the invasive edge. The bioinformatic analysis of the profile of miRNAs and their target mRNAs, as well as the involved signaling pathways, revealed specific differences between invading tumor cells and the rest of the tumor mass [105]. 3D microfluidic cell models of cervical cancer are still under development, and conventional tumor lines (for example, HeLa [106]) are used for this.

## Conclusion

The works which have been published to date on the application of high-throughput methods of genomic analysis to cellular model systems, clearly demonstrate that the combination of these technologies allows to investigate an incredibly wide range of problems and solve issues of different levels of complexity. Nevertheless, when searching the literature, we came across a very modest number of articles on the study of HPV-associated cancer in comparison with epithelial tumors of other etiology and localization. The underlying cause is attributable to the peculiarities of the biology of the object itself, which make it difficult to implement the methods of tissue/cellular modeling in practice, and this, in turn, slows down the development and implementation of new methods of therapy [87]. Today, there is an urgent need to standardize the protocols for creating and using cellular models of primary tumor cells for preclinical trials or personalized diagnostics and therapy, and a need to add a 4^th^ dimension (time) in experiments with three-dimensional cellular systems is being discussed. The great translational potential of the cancer models described above, which are rapidly approaching complete biomimicry, and sharply increased contribution of HPV-associated neoplasias to the structure of overall morbidity and mortality from oncologic pathologies in recent years are expected to lead to wider application of whole-genome profiling methods and a multiomics approach to the analysis of cellular systems of HPV-associated cancer.
